# Influence of Music on Cortisol Levels in Mechanically Ventilated Critically Ill Patients: A Systematic Review

**DOI:** 10.1111/nicc.70475

**Published:** 2026-04-07

**Authors:** Carmen Fernández‐Álvarez, David Zuazua‐Rico, M. Pilar Mosteiro‐Díaz, Alba Maestro‐González

**Affiliations:** ^1^ Hospital Universitario Central de Asturias Oviedo Spain; ^2^ Faculty of Medicine and Health Sciences University of Oviedo Oviedo Spain; ^3^ Instituto de Investigación del Principado de Asturias Oviedo Spain

**Keywords:** artificial, critical care, hydrocortisone, music therapy, nursing, respiration

## Abstract

**Background:**

Music has been proposed as a simple, non‐invasive intervention to modulate stress responses in patients undergoing invasive mechanical ventilation (IMV). Its potential impact on cortisol levels represents a promising approach to mitigating physiological stress in intensive care.

**Aim:**

To examine the effectiveness of music interventions in reducing cortisol levels and, consequently, modulating the stress response in mechanically ventilated patients in intensive care units (ICUs).

**Study Design:**

A systematic review was conducted in accordance with PRISMA 2020 guidelines and registered in PROSPERO (CRD42023409196). Five electronic databases were searched without date restrictions. Eligible studies were randomised controlled trials (RCTs) assessing music interventions in adult patients receiving IMV, with serum cortisol as the primary outcome. Risk of bias was assessed using the Cochrane RoB 2 tool. Owing to substantial heterogeneity, findings were synthesised narratively.

**Results:**

Five RCTs (*n* = 208) met the inclusion criteria. Three reported significant reductions in cortisol following music interventions, while two found no differences. Variability in music type, intervention duration, sedation status and measurement timing contributed to inconsistent results. Risk of bias was low in two studies, whereas three were rated as raising some concerns.

**Conclusions:**

Current evidence on the effect of music on cortisol in mechanically ventilated patients is limited and inconsistent. Findings remain contradictory, preventing firm conclusions about efficacy. Future multicentre trials with standardised protocols and rigorous control of confounders are required.

**Relevance to Clinical Practice:**

This review highlights a major gap in evidence regarding the biological impact of music in IMV patients. Results from five small, heterogeneous RCTs are inconclusive, and some of them are limited by methodological concerns. Thus, current evidence is insufficient to support changes in clinical practice. While music remains a low‐risk intervention of interest, its actual effect on physiological stress has yet to be demonstrated. Large‐scale, high‐quality studies are needed before clinical recommendations can be made.

**Systematic Review Registration:** PROSPERO ID: CRD42023409196.

## Impact Statements

- What is known about the topic
  ○ Critically ill patients receiving invasive mechanical ventilation experience high stress and elevated cortisol levels.
  ○ Elevated cortisol and the use of pharmacological sedation are both associated with adverse clinical outcomes and risks.
  ○ Music therapy has been proposed as a non‐pharmacological intervention to reduce stress.
- What this paper adds
  ○ This systematic review synthesises the current evidence on the effects of music interventions on serum cortisol levels in mechanically ventilated adults.
  ○ The findings show variable results: three studies reported significant reductions in cortisol, while two found no effect.
  ○ The available evidence remains insufficient to support the routine clinical use of music therapy for this purpose.
  ○ The review highlights the need for high‐quality, multicentre trials with standardised protocols to clarify the biological impact of music before clinical recommendations can be made.

## 1 Introduction

Invasive Mechanical Ventilation (IMV) is essential in Intensive Care Units (ICUs), yet it frequently causes discomfort, anxiety and psychological stress. These effects are often managed with sedatives and hypnotics, which may themselves contribute to adverse outcomes [1, 2].

Critically ill patients undergoing IMV experience significant physiological stress that activates the hypothalamic–pituitary–adrenal (HPA) axis, leading to increased cortisol secretion [3, 4, 5, 6]. Although this mechanism is adaptive in responding to acute physiological demands, sustained activation may hinder recovery and worsen prognosis. In ICU settings, illness severity and exposure to invasive procedures (such as intubation, sedation and airway suctioning) further intensify the stress response, increasing the risk of complications and prolonged hospital stays [7, 8, 9, 10].

In healthy individuals, cortisol secretion follows a circadian rhythm. However, in critically ill patients, this pattern is disrupted by persistent stimuli such as pain, respiratory distress and sleep deprivation, leading to chronic activation of the HPA axis and hormonal imbalance [3, 4, 5, 6]. Altered cortisol metabolism and reduced binding proteins contribute to elevated free cortisol concentrations, which may not be adequately reflected in total serum cortisol levels [11].

In this context, stress in patients undergoing IMV is not only an expected consequence but also a frequent manifestation of critical illness. Its origin is multifactorial, involving both physiological factors (such as pain, dyspnoea and hypoxaemia) and psychological factors (such as anxiety, fear and loss of autonomy), which interact and mutually reinforce one another [12, 13, 14]. Additionally, routine clinical procedures, including progressive sedation withdrawal, ventilator weaning and airway suctioning, may intensify discomfort and amplify the stress response [15, 16].

To manage this stress, pharmacological interventions are widely used, including benzodiazepines, opioids and propofol. However, these agents are associated with adverse effects such as respiratory depression, delirium, tolerance and withdrawal syndromes [17, 18]. Moreover, excessive sedation may delay airway extubation and prolong ICU length of stay [19, 20, 21]. Consequently, non‐pharmacological methods have been explored as safer and more sustainable alternatives to reduce stress and improve patient outcomes [19, 20, 21, 22].

Among these, music therapy has gained attention for its potential to modulate stress responses through mechanisms of emotional regulation, distraction and relaxation [23, 24, 25, 26]. Music may influence autonomic nervous system activity and HPA axis responses, potentially reducing stress‐related physiological activation [24, 25, 26]. Various approaches exist, ranging from ambient background music to personalised playlists delivered through headphones, with effectiveness depending on individual preferences, clinical context and mode of delivery [25, 27].

Previous systematic reviews have examined music interventions in critically ill patients and reported benefits in reducing anxiety and improving patient comfort [28, 29]. Although some of these reviews briefly mentioned hormonal findings from isolated trials, they did not systematically evaluate serum cortisol and largely focused on psychological or general physiological outcomes, often in heterogeneous ICU populations. Other relevant reviews, such as de Witte et al. [30], assessed music interventions for stress reduction across a broad range of healthcare settings but did not focus specifically on the biological stress response in mechanically ventilated patients. Furthermore, individual studies frequently cited in the literature, such as those by Leardi et al. [31], were conducted in conscious patients under local anaesthesia, thereby falling outside the scope of IMV.

The present review addresses this gap by providing an updated, focused synthesis of the available evidence. It applies strict inclusion criteria and concentrates on adult patients receiving IMV, aiming to provide a biologically grounded assessment of the impact of music interventions on stress, as measured by serum cortisol levels. By focusing on cortisol—a well‐established biomarker of physiological stress—this review seeks to provide objective data to support the integration of music therapy into critical care practice.

### 1.1 Rationale for the Review

Non‐pharmacological interventions, particularly music, have gained clinical interest due to their safety, accessibility and potential to reduce stress without pharmacological side effects [16, 29, 32]. Music may influence autonomic nervous system activity and cortisol regulation through mechanisms involving emotional processing, distraction and sensory stimulation [33]. In clinical practice, music interventions have taken different forms, from ambient background music to personalised selections delivered through audio devices [28, 34]. Although these strategies have shown benefits in reducing stress and anxiety, their effects vary depending on the degree of individualisation, the clinical setting and the mode of delivery [30].

While most studies have focused on ventilated patients in ICUs [10, 35], some clinical trials have included mechanically ventilated patients in other hospital settings, such as operating theatres, due to shared clinical features (sedation, intubation, mechanical ventilation and heightened stress response), which justify their inclusion in systematic reviews exploring the relationship between IMV, physiological stress and music therapy [31, 36].

### 1.2 Objectives and Review Question

The objective of this systematic review was to evaluate the effectiveness of music interventions in reducing stress, as measured by serum cortisol levels, in adult patients undergoing Invasive Mechanical Ventilation (IMV). Secondary objectives included assessing the effects on sedative use and vital signs as well as identifying characteristics of effective music interventions.

Using the *PICO framework*, the review question is formulated as follows:

- *Population (P)*: Adults undergoing Invasive Mechanical Mentilation (IMV) in hospital settings;
- *Intervention (I)*: Music interventions (ambient music, personalised playlists, or other music therapies);
- *Comparison (C)*: Standard care or no music intervention;
- *Outcome (O)*: Reduction in cortisol levels (primary outcome); secondary outcomes include sedation requirements and vital sign changes.

Are music interventions effective in reducing cortisol levels and modulating the stress response, compared to standard care, in adults undergoing invasive mechanical ventilation?

## 2 Methods

### 2.1 Protocol and Registration

This review was conducted in accordance with the methodological recommendations of the *Cochrane Handbook for Systematic Reviews of Interventions* [37]—specifically, the guidelines on risk‐of‐bias assessment (Chapter 8) and the evaluation and management of heterogeneity (Chapter 10)—and was reported following the PRISMA 2020 statement [38]. The protocol was registered in PROSPERO (ID: CRD42023409196).

**FIGURE 1:**
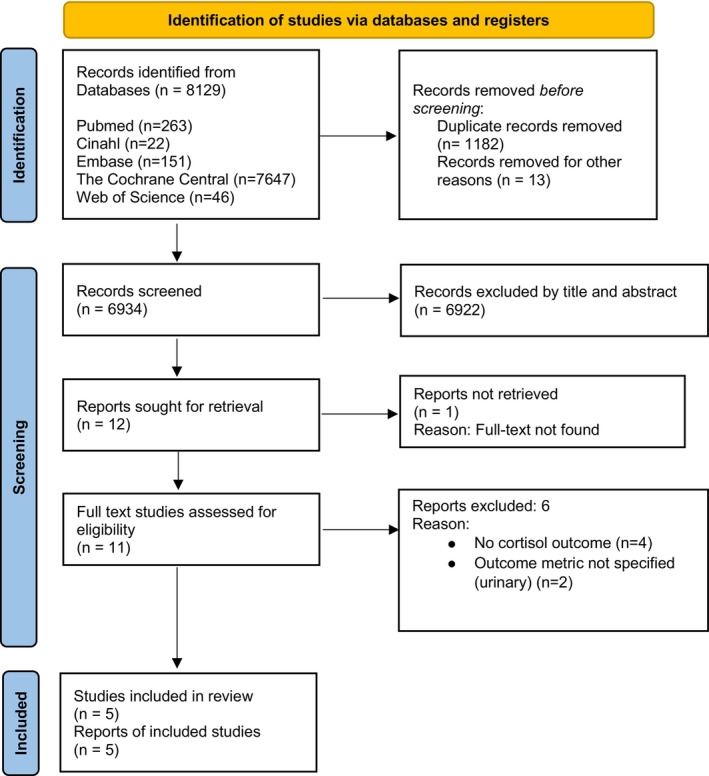
PRISMA 2020 flow diagram for new systematic reviews which included searches of databases and registers.

A meta‐analysis was planned if the included studies demonstrated sufficient clinical and methodological homogeneity in terms of population, intervention, comparator and outcomes. Otherwise, a narrative synthesis would be undertaken to integrate the findings. The study selection process is illustrated in Figure 1, following the PRISMA 2020 flow diagram.

### 2.2 Eligibility Criteria

Studies were included if they met the following criteria: (1) adults undergoing IMV; (2) administration of a music intervention; (3) serum cortisol measured as a primary outcome; and (4) Randomised Controlled Trial (RCT) design.

Exclusion criteria comprised non‐RCT designs, incomplete or missing outcome data, retracted publications and non‐peer‐reviewed sources such as theses, letters or narrative reviews.

### 2.3 Information Sources and Search Strategy

A comprehensive search was conducted in the following databases: the Cochrane Central Register of Controlled Trials (CENTRAL), Embase, PubMed, CINAHL (Cumulative Index to Nursing and Allied Health Literature) and Web of Science. An experienced librarian collaborated with the research team to identify relevant search terms and refine the search strategy. A search strategy combining relevant terms using Boolean operators (AND, OR) was applied. The complete search strings for each database are presented in Table S1.

Only articles published in English and Spanish were considered for inclusion. No restrictions were placed on the publication date, allowing the identification of all potentially relevant studies regardless of when they were published.

In addition to database searches, the reference lists of the included studies and relevant prior systematic reviews were manually screened to identify potentially eligible studies. The search was conducted between 15 March 2023 and 21 June 2024. Retrieved titles were imported into Zotero (v6.0) for initial management and duplicate removal.

### 2.4 Study Selection and Data Extraction

Screening was performed in Rayyan (Qatar Computing Research Institute). Two reviewers independently applied inclusion/exclusion labels and added comments. Disagreements were resolved by consensus or, if necessary, by a third reviewer.

A two‐phase strategy was used to efficiently manage the large volume of records efficiently: first, title screening to exclude clearly irrelevant studies; second, abstract review; and finally, full‐text assessment for definitive inclusion.

Data extracted included: study design, country, sample size, mean age, sedation use, type of music, duration of the intervention and outcomes. Data extraction was conducted using a standardised form and verified by three reviewers. No contact was made with the study authors to obtain additional information.

### 2.5 Risk of Bias and Quality of Evidence Assessment

The methodological quality of the included trials was independently assessed by two reviewers using the Cochrane RoB 2 tool [37, 39], applying the specific variants for parallel‐group and crossover trials. The five standard domains were assessed according to the official decision rules. Notably, open‐label designs were not considered high risk regarding outcome measurement, as serum cortisol is an objective biochemical marker unlikely to be influenced by lack of blinding. In accordance with RoB2 guidance, the absence of blinding alone does not lead to a ‘high risk’ judgement when the outcome is objective and not prone to detection bias. Conversely, lack of detail on randomisation or allocation concealment was rated as ‘some concerns’. Disagreements were resolved by consensus. Overall assessments (‘low risk’, ‘some concerns’ or ‘high risk’) followed the RoB‐2 algorithm. Risk‐of‐bias graphs were generated using the Risk‐Of‐Bias VISualization tool (robvis).

### 2.6 Outcomes and Data Synthesis

The primary outcome was the change in serum cortisol levels (μg/dL or nmol/L) in response to the intervention. Secondary outcomes, where reported, included variations in sedative use and physiological parameters. The data synthesis plan was prespecified in the study protocol. A meta‐analysis was planned if the included studies showed sufficient clinical and methodological homogeneity (population, intervention, comparator, outcomes). Otherwise, a narrative synthesis would be used to integrate the findings.

## 3 Results

### 3.1 Search Results

A total of 8129 records were identified across the databases (PubMed, CINAHL, Embase, Cochrane Library and Web of Science). After removal of 1182 duplicates and 13 records excluded for other reasons, 6934 titles and abstracts were screened. Of these, 6922 were excluded, leaving 12 reports for retrieval. One could not be retrieved, resulting in 11 studies being assessed in full text for eligibility. At this stage, six reports were excluded (reasons detailed in Table S2), primarily for not reporting cortisol as an outcome (*n* = 4) or for measuring urinary rather than serum cortisol (*n* = 2). Ultimately, five RCTs met the inclusion criteria and were incorporated into the narrative synthesis (Figure 1). As prespecified in the protocol, a meta‐analysis was not conducted due to substantial clinical and methodological heterogeneity among the included studies. Instead, findings were integrated narratively.

### 3.2 Characteristics of the Included Studies

Table 1 summarises the main characteristics of the included studies, all of which were RCTs (*n* = 5). One study employed a crossover design. All were single‐centre trials, conducted in Canada (*n* = 2) [40, 41], the United States (*n* = 1) [42], Taiwan (*n* = 1) [43] and India [44]. The studies were published between 2007 and 2017.

**TABLE 1:** Characteristics of the included studies.

Reference	Country	Study design	Population characteristics									Intervention characteristics			Outcomes			
			Gender		Age distribution		Sample size		Type of population	Sedation	Music style	Interventions	Time	Length of the music	Cortisol baseline		Cortisol Post‐test	
			Male	Female	Control	Music	Control	Music							Control	Music	Control	Music
Beaulieu‐Boire et al. 2013	Canada	Randomised controlled crossover trial	32	17	61 ± 3^a^	63 ± 3^b^	49	49	ICU, mechanical ventilation, adults, haemodynamically stable, SAS 3–4	Propofol, midazolam	Classical music	2	10 and 20 h	60 min	741 ± 71 (nmol/L)	815 ± 126 (nmol/L)	746 ± 68 (nmol/L)	727 ± 98 (nmol/L)
Chlan et al. 2007	United States	Randomised controlled trial	4	6	64.9 ± 7.8		5	5	ICU, mechanical ventilation, adults, central venous access, haemodynamically stable, normal renal function, haemoglobin and haematocrit, no steroid treatment	No	Classical music	1	5.40 h	60 min	580 ± 20.9 (μg/L)	920 ± 33.4 (μg/L)	700 ± 25.2 (μg/L)	820 ± 29.8 (μg/L)
Lee et al. 2017	Taiwan	Randomised controlled trial	37	48	59.52 ± 8.37	59.46 ± 9.87	44	41	ICU, mechanical ventilation, adults, haemodynamically stable, no steroid treatment	No	Election	1	16 h	30 min	8.34 ± 1.73 (μg/L)	8.16 ± 2.25 (μg/L)	8.41 ± 1.95 (μg/L)	8.01 ± 1.97 (μg/L)
Migneault et al. 2004	Canada	Randomised clinical trial	0	30	52.2 ± 9.1	46.3 ± 12.1	15	15	Adults, abdominal hysterectomy, hysterosalpingo‐oophorectomy or salpingo‐oophorectomy, general anaesthesia, mechanical ventilation, no steroid treatment	Propofol, isoflurane	Election	1	8–15 h	Duration of surgery	351 ± 148 (nmol/L)	383 ± 181 (nmol/L)	871 ± 147 (nmol/L)	817 ± 199 (nmol/L)
Kar et al. 2015	India	Randomised clinical trial	NA	NA	NA	NA	17	17	Adult, cardiac surgery under cardiopulmonary bypass (CPB), general anaesthesia, mechanical ventilation	Fentanyl, thiopentone, midazolam, isoflurane, vecuronium	Raga *Darbari*	1	30 min induction until the end of surgery	Duration of surgery	211.99 ± 39.01 (nmol/L)	127.78 ± 31.82 (nmol/L)	528.11 ± 149.48 (nmol/L)	264.59 ± 53.78 (nmol/L)

Abbreviation: NA: not applicable.

^a^ Group A.

^b^ Group B.

### 3.3 Sample Size

Sample sizes varied considerably. Beaulieu‐Boire et al. [41] enrolled 49 participants in a randomized crossover study (24 control, 25 intervention); Chlan et al. [42] included 10 patients (5 per group); Lee et al. [43] had the largest sample with 85 participants (44 control, 41 intervention); Migneault et al. [40] included 30 patients (15 per group); and Kar et al. [44] included 34 patients (17 per group).

All participants were haemodynamically stable adults receiving IMV. In the trials by Chlan et al. [42] and Lee et al. [43], patients were conscious during the intervention. Beaulieu‐Boire et al. [41] included sedated patients with a Riker Sedation‐Agitation Scale (SAS) score of 3 or 4. In contrast, Migneault et al. [40] and Kar et al. [44] focused on patients under general anaesthesia in a perioperative setting. Migneault et al. [40] focused exclusively on women undergoing specific gynaecological procedures (abdominal hysterectomy, hysterosalpingo‐oophorectomy or salpingo‐oophorectomy) whereas Kar et al. [44] included patients undergoing cardiac surgery.

The sex distribution showed a predominance of female participants: Chlan et al. [42] included six women and four men; Lee et al. [43] reported 48 women and 37 men; while Migneault et al. [40] included only women. Kar et al. [44] did not report the sex distribution for their 34 participants. The mean age across studies ranged from 40 to 64 years.

### 3.4 Music Intervention

The five studies varied in design and implementation of the music intervention. Beaulieu‐Boire et al. [41] used a crossover design in which participants were exposed to both conditions: music and control (silent‐headphones). The remaining four studies used a parallel‐group format, with control groups either wearing silent headphones [40, 43, 44] or resting without auditory input [42].

Regarding music content, Beaulieu‐Boire et al. [41] played 10 classical pieces selected by a musicologist. Similarly, Kar et al. [44] employed a standardised intervention consisting of the Indian classical raga *Darbari*. In contrast, the other studies allowed patients to choose their preferred music. In Chlan et al. [42], all participants selected classical music. Lee et al. [43] reported patient selections included Chinese classical music, religious pieces, Western classical music and nature sounds. Migneault et al. [40] offered a broader catalogue, including classical, jazz, new age and popular piano music.

Session duration varied: Beaulieu‐Boire et al. [41] and Chlan et al. [42] used 60‐min sessions; Lee et al. [43] applied a 30‐min intervention; Kar et al. [44] delivered music continuously, beginning 30 min before induction and continuing throughout the entire surgical procedure; and Migneault et al. [40] also played music continuously for the full duration of surgery. Session timing also differed across studies, ranging from early morning to afternoon.

Although the methodological approaches varied, most interventions relied on passive listening through headphones, providing consistent delivery and minimising environmental interference. Only one study [42] used music without headphones, employing quiet rest as its control condition.

Three trials [41, 43, 44] demonstrated statistically significant reductions in cortisol following music interventions. Beaulieu‐Boire et al. [41] observed a significant post‐intervention decrease in cortisol levels (*p* = 0.02), with a more pronounced reduction among patients exposed to music (*p* < 0.001). No significant changes were found in vital signs (heart rate, blood pressure, respiratory rate) although a non‐significant trend towards reduced narcotic use was noted in the intervention group. Similarly, Lee et al. [43] reported statistically significant differences in cortisol levels both post‐intervention and in pre–post comparisons between the intervention and control groups (*p*‐values ranging from 0.02 to < 0.001). Kar et al. [44] likewise found a statistically significant reduction in cortisol levels, with approximately 30% lower levels in the music group than in the silent headphone control group (*p* < 0.05).

In contrast, Chlan et al. [42] and Migneault et al. [40] did not find significant between‐group differences in cortisol concentrations. Nevertheless, Chlan et al. [42] observed a within‐group reduction in cortisol among participants exposed to the music condition, suggesting a possible intra‐individual effect.

### 3.5 Risk of Bias Assessment

The risk of bias assessment, conducted using the *Cochrane Risk of Bias 2 (RoB 2)* tool, revealed that two studies (Beaulieu‐Boire et al. [41] and Lee et al. [43]) presented a low risk of bias across all domains.

The remaining three studies were judged as raising *some concerns*. The study by Migneault et al. [40] was limited by insufficient detail regarding sequence generation and allocation concealment. Similarly, the pilot trial by Chlan et al. [42] was considered problematic due to limited information on randomisation procedures and its small sample size. The study by Kar et al. [44] was also classified as having ‘some concerns’, primarily because of the absence of reporting on baseline demographic distribution (sex), lack of detail on allocation concealment during randomisation and the absence of a publicly available preregistered protocol.

**FIGURE 2:**
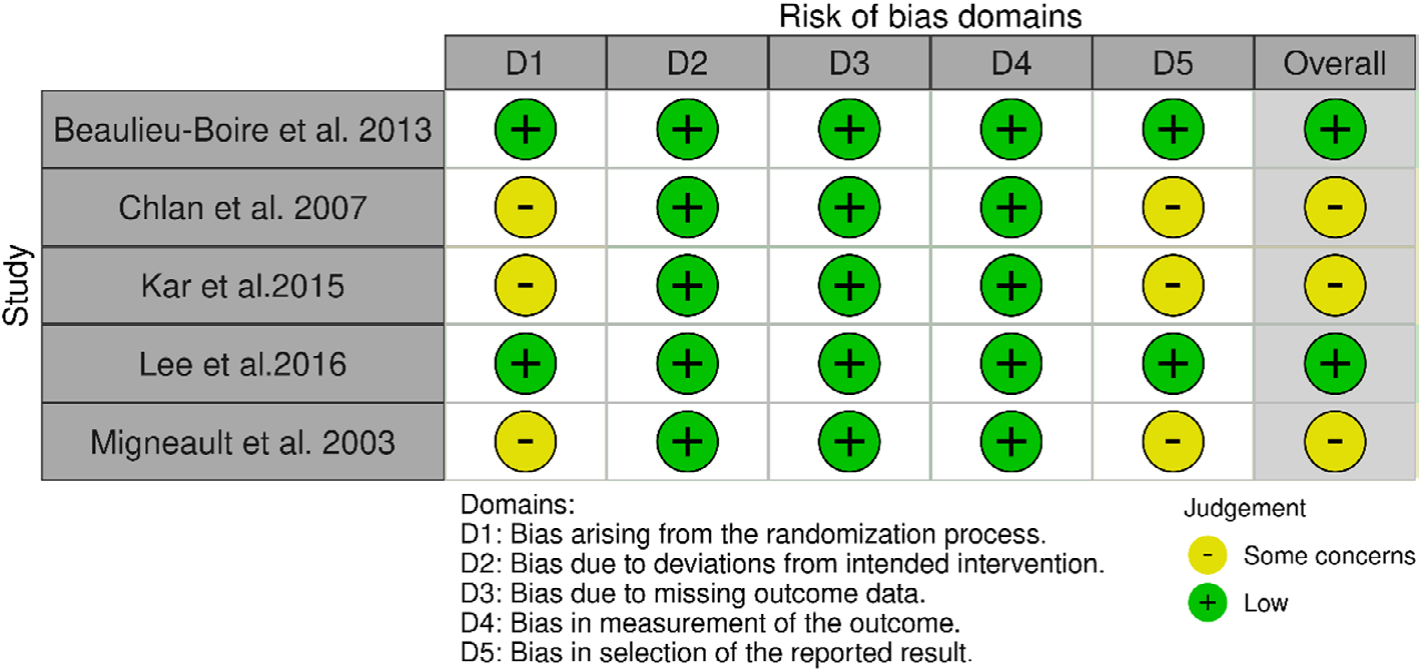
The Risk of Bias (RoB 2.0) assessment of the included studies.

No study was rated as high risk in any domain. Figure 2 presents a summary of domain‐level assessments and the overall risk of bias judgements.

### 3.6 Effect of Music Therapy on Cortisol Levels

The effects of music therapy on cortisol levels in patients undergoing IMV varied across the included studies. Beaulieu‐Boire et al. [41] observed a significant reduction in cortisol levels following the music intervention. In the group that listened to music (M+), cortisol levels decreased from 815 ± 126 nmol/L to 727 ± 98 nmol/L (*p* = 0.02), whereas no significant change was observed in the control group (M−), whose levels shifted from 741 ± 71 nmol/L to 746 ± 68 nmol/L (*p* = 0.83). The mean individual difference was −85 ± 39 nmol/L in the music group versus +5 ± 24 nmol/L in the placebo group (*p* = 0.05). Furthermore, a greater number of patients were classified as ‘biological responders’ following the music intervention (39 vs. 24, *p* = 0.005).

Lee et al. [43] also reported statistically significant differences. The intervention group exhibited a pre–post reduction of −0.05 ± 0.10 μg/L (equivalent to approximately −1.38 nmol/L), compared to a slight increase in the control group (+0.20 ± 0.11 μg/L). The between‐group comparison was significant (*p* = 0.03). The post‐intervention cortisol level was lower in the music group (8.21 ± 0.10 μg/L) compared to the control group (8.46 ± 0.11 μg/L, *p* = 0.02).

Kar et al. [44] similarly demonstrated significant reductions in cortisol following music exposure. Cortisol levels measured 30 min after induction and after aortic cross‐clamping were markedly lower in the music group (127.78 ± 31.82 nmol/L and 264.59 ± 53.78 nmol/L, respectively) compared with the silent headphone control group (211.99 ± 39.01 nmol/L and 528.11 ± 149.48 nmol/L; both *p* < 0.05). These reductions coincided with lower intraoperative requirements for fentanyl, propofol and vecuronium, findings consistent with decreased physiological stress.

By contrast, Chlan et al. [42] did not find statistically significant differences between groups, although a decreasing trend was observed within the intervention group. Cortisol levels in the music group declined from 920 nmol/L (33.4 μg/dL) at baseline to 820 nmol/L (29.8 μg/dL) at 60 min, while levels in the resting group increased from 580 nmol/L (21.0 μg/dL) to 700 nmol/L (25.2 μg/dL). Although the Friedman test did not reveal overall significance, the pattern suggests a possible intra‐individual reduction in the music group.

In contrast, Migneault et al. [40] did not report significant differences in cortisol levels between the music and non‐music groups. In the non‐music group (NM), cortisol values were T1: 351 ± 148 nmol/L; T2: 334 ± 150 nmol/L; T3: 871 ± 147 nmol/L; and T4: 1050 ± 239 nmol/L. In the music group (M), values were similar: T1: 383 ± 181 nmol/L; T2: 359 ± 157 nmol/L; T3: 817 ± 199 nmol/L; and T4: 903 ± 204 nmol/L. Although increases over time were noted, likely due to surgical stress, no statistically significant differences were observed between the groups.

## 4 Discussion

This systematic review evaluated the effect of music interventions on cortisol levels in critically ill patients and those undergoing invasive procedures. Collectively, the included studies encompassed heterogeneous populations, ranging from mechanically ventilated ICU patients to individuals undergoing cardiac surgery. While this diversity highlights the broad applicability of music interventions, it also complicates direct comparison.

Our findings reveal a promising but inconsistent biological signal. Three of the five trials reported statistically significant reductions in cortisol, notably the study by Kar et al. [44], which demonstrated substantial intraoperative decreases even under general anaesthesia. Conversely, Chlan et al. [42] and Migneault et al. [40] found no significant differences. It is noteworthy that both negative studies were rated as raising *some concerns* in the RoB 2 assessment, whereas two of the positive trials (Beaulieu‐Boire et al. [41] and Lee et al. [43]) were judged to be at low risk of bias. This pattern suggests that rigorous methodologies may be better able to detect the subtle physiological effects of music.

The overall quality of evidence was moderate. The risk of bias assessment identified concerns primarily related to incomplete reporting of randomisation and the inherent difficulty of blinding. However, the most significant limitation was the marked clinical and methodological heterogeneity. Variations in music selection (researcher‐selected vs. patient‐preferred), intervention timing and pharmacological contexts (sedation vs. general anaesthesia) likely acted as confounders. For instance, the use of opioids and sedatives—potent modulators of the stress response—was not consistently controlled, potentially masking the effects of music. Furthermore, the use of silent headphones in control groups in four studies may have introduced an acoustic isolation effect, making it difficult to distinguish the specific impact of music from general noise reduction.

Our results refine and extend previous syntheses. While Bradt and Dileo [28] established the efficacy of music for subjective outcomes like anxiety, their physiological analysis was limited. More recent reviews by Papathanassoglou et al. [45] and Umbrello et al. [29] have suggested a potential benefit for cortisol but, like us, cautioned against strong conclusions due to heterogeneity. Our review corroborates this complexity: the physiological plausibility of music to modulate the HPA axis is supported by the majority of our included trials (3/5), but clinical consistency remains elusive due to the lack of standardised protocols.

In conclusion, while the hypothesis that music modulates the endocrine stress response is coherent and supported by positive findings in higher quality trials, the evidence remains insufficient for definitive clinical recommendations. Future research should prioritise multicentre designs with standardised protocols that rigorously control for pharmacological confounders and clearly differentiate the effects of music from those of acoustic isolation.

## 5 Implications for Clinical Practice

This systematic review suggests that music therapy may hold potential as a non‐pharmacological complementary intervention in critically ill patients undergoing IMV. From a clinical perspective, music interventions could contribute to reducing the activation of the HPA axis, as evidenced by the significant decreases in cortisol levels observed in three of the included studies [41, 43, 44].

While current evidence is insufficient to support a mandatory standard of care, incorporating music therapy as a low‐risk adjuvant strategy in the ICU could enhance patient comfort, support humanised care and optimise the therapeutic environment. For such implementation to be effective, safe and reproducible, it is essential to develop standardised protocols defining music selection, session duration, timing of administration and patient profiles.

In addition, nursing staff should receive specific training, as recommended by clinical guidelines [17, 32], which highlight the use of therapeutic music libraries or digital applications as effective tools to ensure intervention fidelity.

Although variability among studies prevents definitive recommendations, preliminary evidence supports the use of patient‐preferred or culturally appropriate relaxing music delivered via headphones in clinically controlled settings. This strategy could form part of a multimodal, patient‐centred approach to stress management in critically ill patients.

## 6 Limitations

This systematic review presents certain limitations that should be considered when interpreting its findings. First, although both English and Spanish‐language articles were included, studies published in other languages may have been overlooked. Second, despite an initially broad search strategy, only five RCTs met the strict inclusion criteria, reflecting a relatively limited evidence base and constraining the generalisability of the results. Third, methodological variability among the included studies (such as differences in music selection, timing of the intervention and patient sedation status) precluded conducting a meta‐analysis and limited direct comparisons. Lastly, as grey literature and unpublished data were not included, there remains a risk of publication bias.

## 7 Conclusion

Music therapy may contribute to reductions in cortisol levels in specific clinical contexts, providing a promising yet preliminary signal. However, methodological heterogeneity and small sample sizes constrain the robustness of the current evidence. Despite these limitations, music therapy appears to be a safe and feasible complementary intervention. Future multicentre randomised controlled trials with standardised protocols are required to validate these findings and to elucidate the precise role of music in modulating the endocrine stress response.

## Funding

This work was supported by the Beca Investiga 2023, granted by the Sociedad Española de Enfermería Intensiva y Unidades Coronarias (SEEIUC).

## Ethics Statement

The authors have nothing to report.

## Consent

The authors have nothing to report.

## Conflicts of Interest

The authors declare no conflicts of interest.

## Supporting information


**Table S1:** Search strategy by database (until 21 June 2024).

**Table S2:** Full‐text articles excluded with reasons (PRISMA 2020).

## Data Availability

The data supporting the findings of this study are available within the article and its Supporting Information.
