# Supplementary material for: Influence of Music on Cortisol Levels in Mechanically Ventilated Critically Ill Patients: A Systematic Review
Source: Nurs Crit Care. 2026 Apr 7;31(3):e70475. doi: 10.1111/nicc.70475 (PMC13058171; doi:10.1111/nicc.70475)
Supplement: Supplementary file 1 — Table S1: Search strategy by database (until 21 June 2024). [file NICC-31-0-s002.docx]

**Table S1. Search Strategy by Database (until 21 June 2024).**

This table details the complete search strategies used across all databases, including MeSH terms, keywords, and Boolean operators. The number of records retrieved from each database is also reported.

| **Database** | **Search Strategy** | **Results** |
| --- | --- | --- |
| **PUBMED** | ((((((("Critical Care"[Mesh] OR "Critical Care Nursing"[Mesh] OR "Critical Care Outcomes"[Mesh] OR "Critical Pathways"[Mesh] OR critical care[Title/Abstract] OR critical care nursing[Title/Abstract] OR critical care outcomes[Title/Abstract] OR critical pathways[Title/Abstract]))) OR ((General Surgery[Title/Abstract]) OR (General Surgery[MeSH Terms]))) AND (((anesthesia[MeSH Terms]) OR (Anesthesia[Title/Abstract])) OR ((("Conscious Sedation"[Mesh]) OR (Moderate Sedation[Title/Abstract])) OR (sedation[Title/Abstract])))) AND ((("respiration, artificial"[MeSH Terms]) OR (respiration artificial[Title/Abstract])) OR (mechanical* ventila*[Title/Abstract]))) OR (cortisol[Title/Abstract])) AND (((((Music[Title/Abstract]) OR ("music"[MeSH Terms])) OR (music therapy[MeSH Terms])) OR (Music Therapy[Title/Abstract])) OR (music*[Title/Abstract])) | 263 |
| **CINAHL** | (((MH “Conscious Sedation”) OR (MH “Sedation”) OR (MH “Respiration, Artificial”)) AND ((MH “Critical Care Nursing”) OR (MH “Critical Care”)) AND ((MH “Music Therapy”) OR (MH “Music”))) | 22 |
| **EMBASE** | ((“intensive care nursing”/exp OR “intensive care unit”/exp) AND ((“conscious sedation”/exp OR “sedation”/exp OR “artificial ventilation”/exp) OR (“anesthetic agent”/exp OR “anesthesia”/exp)) AND (“music”/exp OR “music therapy”/exp)) | 151 |
| **THE COCHRANE LIBRARY** | Critical Care Nursing (Title, Abstract, Keyword) AND Conscious Sedation (Title, Abstract, Keyword) OR respiration artificial (Title, Abstract, Keyword) AND music therapy (Title, Abstract, Keyword) OR music (Title, Abstract, Keyword) - (Word variations have been searched) | 7647 |
| **WOS** | (TI=(intensive care nursing))AND (TI=(conscious sedation) OR TI=(sedation) OR TI=(artificial ventilation) OR (TI=(anesthesia)) AND TI=(music) OR TI=(music therapy)) | 46 |
| **Total** |  | **8129** |

ICU = intensive care unit; MeSH = Medical Subject Headings
