# Supplementary material for: Influence of Music on Cortisol Levels in Mechanically Ventilated Critically Ill Patients: A Systematic Review
Source: Nurs Crit Care. 2026 Apr 7;31(3):e70475. doi: 10.1111/nicc.70475 (PMC13058171; doi:10.1111/nicc.70475)
Supplement: Supplementary file 2 — Table S2: Full‐text articles excluded with reasons (PRISMA 2020). [file NICC-31-0-s001.docx]

**Supplementary Table S2. Full-text articles excluded with reasons (PRISMA 2020).**

This table summarizes the full-text studies that were reviewed but excluded from the final synthesis, including the specific reasons for exclusion according to PRISMA 2020 guidelines.

| **First autor (year)** | **Journal** | **Reason for Exclusion** |
| --- | --- | --- |
| Chlan L (1998) | *Heart & Lung* | Did not measure cortisol. |
| Gambrell K et al. (2016) | *Critical Care Medicine* | Did not measure cortisol. |
| Lin Han et al. (2010) | *Journal of Clinical Nursing* | Did not measure cortisol. |
| Luis et al. (2019) | Global Cardiology Science & Practice | Did not measure cortisol. |
| Chlan LL et al. (2013) | *Intensive and Critical Care Nursing* | Measured urinary cortisol. |
| Hu R et al. (2015) | *Critical Care* | Measured urinary cortisol. |
